# Gain-of-Function Mutations in the Toll-Like Receptor Pathway: TPL2-Mediated ERK1/ERK2 MAPK Activation, a Path to Tumorigenesis in Lymphoid Neoplasms?

**DOI:** 10.3389/fcell.2016.00050

**Published:** 2016-05-26

**Authors:** Simon Rousseau, Guy Martel

**Affiliations:** Meakins-Christie Laboratories, Department of Medicine, McGill University, and McGill University Health Centre Research InstituteMontreal, QC, Canada

**Keywords:** MYD88, blood cancer, hematologic malignancies, NFκB, lymphocytes, B cells

## Abstract

Lymphoid neoplasms form a family of cancers affecting B-cells, T-cells, and NK cells. The Toll-Like Receptor (TLR) signaling adapter molecule MYD88 is the most frequently mutated gene in these neoplasms. This signaling adaptor relays signals from TLRs to downstream effector pathways such as the Nuclear Factor kappa B (NFκB) and Mitogen Activated Protein Kinase (MAPK) pathways to regulate innate immune responses. Gain-of-function mutations such as MYD88[L265P] activate downstream signaling pathways in absence of cognate ligands for TLRs, resulting in increased cellular proliferation and survival. This article reports an analysis of non-synonymous somatic mutations found in the TLR signaling network in lymphoid neoplasms. In accordance with previous reports, mutations map to MYD88 pro-inflammatory signaling and not TRIF-mediated Type I IFN production. Interestingly, the analysis of somatic mutations found downstream of the core TLR-signaling network uncovered a strong association with the ERK1/2 MAPK cascade. In support of this analysis, heterologous expression of MYD88[L265P] in HEK293 cells led to ERK1/2 MAPK phosphorylation in addition to NFκB activation. Moreover, this activation is dependent on the protein kinase Tumor Promoting Locus 2 (TPL2), activated downstream of the IKK complex. Activation of ERK1/2 would then lead to activation, amongst others, of MYC and hnRNPA1, two proteins previously shown to contribute to tumor formation in lymphoid neoplasms. Taken together, this analysis suggests that TLR-mediated ERK1/2 activation via TPL2 may be a novel path to tumorigenesis. Therefore, the hypothesis proposed is that inhibition of ERK1/2 MAPK activation would prevent tumor growth downstream of MYD88[L265]. It will be interesting to test whether pharmacological inhibitors of this pathway show efficacy in primary tumor cells derived from hematologic malignancies such as Waldenstrom's Macroglobulinemia, where the majority of the cells carry the MYD88[L265P] mutation.

## Overview: the toll-like receptor (TLR) signaling adapter MYD88 is the most frequently mutated gene in lymphoid neoplasms

The adven Next Generation Sequencing has made available rich data sources to better understand biological processes. In the field of cancer, it is now possible to obtain a better idea of the mutations landscape of specific types of tumors. Analysis of this information can yield a greater insight into the pathogenesis of specific disorders. In this Hypothesis and Theory article, we have mined the Catalog Of Somatic Mutations In Cancer (COSMIC) to better understand the process of cellular transformation of lymphocytes (lymphoid neoplasms). We focused on the Toll-Like Receptor (TLR) signaling pathway as previous evidences had highlighted an important role in B cell transformation for MYD88, one of its main downstream signaling adaptor.

**Figure 1:**
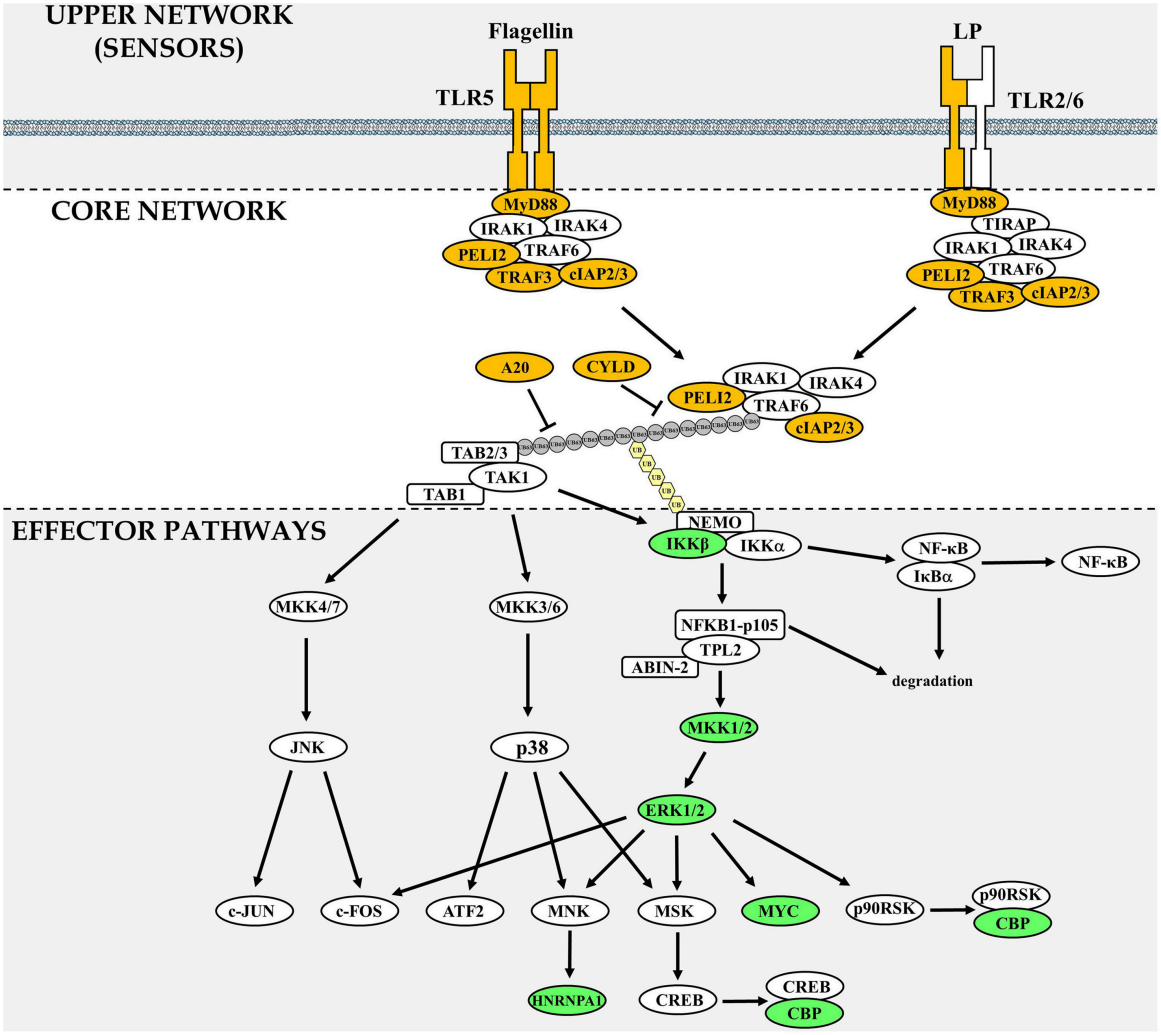
**Components of the TLR signaling network frequently mutated in lymphoid neoplasms**. The TLR signaling network is divided in three sections: upper network, which comprises sensors, core network that is common to MYD88-dependent signaling and downstream effector pathways. Gene products that are found frequently mutated in lymphoid neoplasms are highlighted in yellow (upper and core network) or green (downstream signaling effector pathways). In the core network, the gray circles represent ubiquitin chains linked via Lysine 63 and the pale yellow hexagons represent linear ubiquitin chains. A pathway comprising all 77 network components investigated can be found in Supplementary Figure 1.

MYD88 is mutated in 22% of lymphoid tumor samples according to the COSMIC database (Sanger institute, UK; Forbes et al., 2014) (Table 1). This critical signaling adaptor normally relays signals from TLRs to downstream effector pathways such as the Nuclear Factor kappa B (NFκB) and Mitogen Activated Protein Kinase (MAPK) pathways to regulate innate immune responses (Kawai and Akira, 2010). Gain-of-function mutations such as MYD88[L265P] activate downstream signaling pathways in absence of cognate ligands for TLRs, resulting in increased cellular proliferation, and survival (Yang et al., 2013; Ansell et al., 2014; Avbelj et al., 2014). The medical and scientific literature investigating MYD88 role in cancer focuses on NFκB activation (Treon et al., 2012; Yang et al., 2013). However, TLR-mediated activation of MYD88 also leads to the activation of other signaling pathways such as ERK1/2, p38 MAPK, and JNK (Figure 1). The contribution of these other effector signaling pathways to tumor formation in the context of TLR-activation has been largely overlooked and deserves closer attention. In order to formulate a testable hypothesis on the identity of the TLR effector pathway(s) driving the tumorigenic process, we first investigated the frequency of mutations of TLR signaling components in lymphoid neoplasms.

**Table 1:** **Mutation frequency of TLR-signaling network components in lymphoid neoplasms**.

**Id**.	**Alt Id**	**No of mutations**	**Samples tested**	**% of mutations**	**Most common non-silent mutation(s)**
MYD88	MYD88	1754	7854	22.3	1584[L265P]
A20	TNFAIP3	214	3073	7.0	25[whole gene del]
CBP	CREBBP	174	2605	6.7	12[R1446H]
cMyc	MYC	40	1500	2.7	6[F138S]
ciap2	BIRC3	110	4173	2.6	12 [whole gene del]
MKK1	MAP2K1	40	1694	2.4	4[C121S]
TLR2	TLR2	8	1415	0.6	4[D327V]
CYLD	CYLD	10	1832	0.5	6[whole gene del]
TRAF3	TRAF3	8	1673	0.5	2[whole gene del]
IKKβ	IKBKB	8	1727	0.5	8[K171E]
TLR5	TLR5	5	1415	0.4	2[N96K]
PELI2	PELI2	5	1415	0.4	2[R154W]
hnRNPA1	HNRNPA1	4	1496	0.3	[S22T]; [E9K]; [T138S]
ERK2	MAPK1	4	1508	0.3	[D162N];[D291G];[R124H];[Y316F]
MKK2	MAP2K2	4	1589	0.3	2[Q60P]
ciap1	BIRC2	3	1415	0.2	
TLR4	TLR4	3	1415	0.2	
TLR8	TLR8	3	1415	0.2	
MKK4	MAP2K4	3	1460	0.2	
TLR6	TLR6	2	1415	0.1	
pellino3	PELI3	2	1415	0.1	
TAB3	TAB3	2	1415	0.1	
ABIN1	TNIP1	2	1415	0.1	
HOIP	RNF31	2	1415	0.1	
SMAD6	SMAD6	2	1415	0.1	
TTP	ZFP36	2	1415	0.1	
TAB2	TAB2	2	1415	0.1	
TLR1	TLR1	2	1415	0.1	
ERK1	MAPK3	2	1423	0.1	
IRAK4	IRAK4	2	1423	0.1	
TPL2	MAP3K8	2	1423	0.1	
JNK2	MAPK9	2	1423	0.1	
MNK2	MKNK2	2	1423	0.1	
IRAK1	IRAK1	2	1508	0.1	

*Genes with a mutation frequency >0.25 are in Yellow (upper and core TLR network) or Green (downstream effector pathways)*.

*List of genes analyzed with: 1 mutation: TOLLIP, ATF2, CREB, SHARPIN, ECSIT, ELK1, MSK1, TBK1, TLR7, TAX1BP1, TAK1, MKK7, JNK1, p38α, IKKα, MSK2, IKKε, MAPKAPK2, PELI1*.

*No mutation: ABIN2, TIRAP, TRAF6, TAB1, p105, MKK6, JNK3, p38β, ERK5, NEMO, IκBα, IκBβ, NFκB(p65), cJun, cFOS, eiF4E, HSP27, UBC13, Uev1a, TANK, IRAKM, TIFA, SCF-βTRCP, HOIL1, hnRNPA0, MKK3, OTUL*.

## Analysis of somatic mutations found in the TLR signaling network in lymphoid neoplasm

### Overview of the TLR signaling network

The TLR family has 10 members in humans (Carpenter and O'Neill, 2009). Following dimerization, TLRs bind different adaptor molecules through their Toll/IL-1 receptor domain (TIR). The family can be sub-divided between receptors signaling through the adaptors MYD88 (TLR1, TLR2, TLR4, TLR5, TLR6, TLR7, TLR8, and TLR9) or TRIF (TLR3 and TLR4). MYD88 mediates the sequential recruitment of IL-1R-associated protein kinases (IRAK) (Muzio et al., 1997; Wesche et al., 1997), TNF-receptor-associated factor 6 (TRAF6) (Cao et al., 1996) and TGF-β Activated Kinase (TAK1) (Ninomiya-Tsuji et al., 1999; Lee et al., 2000). This signaling cascade leads to the activation of four major signaling pathways: the NFκB pathway and the three MAPK pathways, ERK1/2, JNK, and p38 MAPK (Figure 1). The TRIF adaptor, which can be recruited directly to TLR3, or indirectly via TRAM to TLR4, lead to the production of type I interferons via the activation of the IKK family members IKKε and TBK1 and the phosphorylation of interferon response factors (IRFs) (Yamamoto et al., 2002). TRIF can also mediate NFκB activation via Pellino 1 and RIP1 (Meylan et al., 2004; Chang et al., 2009).

### Non-silent somatic mutations in the TLR pathway

Based on the current literature a TLR signaling network containing 79 distinct molecules involved in MYD88-signaling was assembled. A proposed network organization of these molecules in TLR signaling is illustrated in Supplementary Figure 1 (Oda and Kitano, 2006; Padwal et al., 2014). Each of these molecules was investigated for the presence of non-synonymous somatic mutations in lymphoid neoplasms using data extracted from the COSMIC database (Table 1) (Forbes et al., 2014). The search parameters used were: Tissue selection [Haematopoietic and Lymphoid tissue]; Subtissue selection [Include All]; Histology Selection [Lymphoid neoplasm]; subhistology selection [Include All]. The search results were updated to the most recent values at the end January 2016. To determine the more likely pathway members contributing to tumorigenesis, a mutation frequency threshold was set based on a study looking for oncogenic driver mutations in Chronic Lymphocytic Leukemia (CLL) (Wang et al., 2011). In that study, 88 Tumor samples were studied by exome and whole genome sequencing. Oncogenic drivers were defined as genes having mutation rates significantly higher than the background taking into account sequence composition. The gene identified with the lowest mutation frequency deemed significant was ERK2 (MAPK1). In the gene set investigated in the current analysis, ERK2 was found mutated 4 times out of 1508 samples (Table 1). Therefore, as mutations for ERK2 were deemed significant oncogenic drivers in CLL and that the role of the ERK1/2 MAPK pathway in tumor growth of B cells is well established (Platanias, 2003; Rickert, 2013), the threshold of significance in this analysis was set at 0.25%.

### Distribution of mutations in the TLR signaling network

The TLR network architecture takes the shape of an hourglass, with multiple TLRs feeding into a core signaling module (MYD88-TRAF6-TAK1), before re-expansion downstream of the activation of TAK1 (Figure 1). When examining mutations identified in the TLR-network above the 0.25% threshold (Table 1), 2 are found in the upper part of the hourglass network (TLR2, and TLR5; 0.9% overall mutation frequency), 6 in the core network (MYD88, A20, ciap2, CYLD, TRAF3, and Pellino 2; 33.4% overall mutation frequency) and 7 in the downstream-activated signaling pathways (CBP, MYC, MKK1, IKKβ, hnRNPA1, MKK2, and ERK2; 13.2% mutation rate). Mutations that lead to activation of the upper part of the TLR network (TLR + core signaling components) account for more than a third of mutations found in lymphoid neoplasms. This assumes that all these mutations only target TLR-signaling, which is unlikely to be the case as regulators of multiple pathways such as CYLD and A20 would impact other pathways like the TNFR-activated pathway (Kovalenko et al., 2003; Trompouki et al., 2003; Shembade et al., 2010) or BAFF-R signaling in the case of ciap2 (Gardam et al., 2011). But even when removing these components from the equation more than a quarter of the mutations would be hypothesize to favor enhanced signaling of the global TLR signaling network. Looking at molecules assigned to effectors of TLR signaling (bottom part of the hourglass) a striking observation is that all of these molecules can be linked to ERK1/2 MAPK signaling (Figure 1). As was the case for ciap2, CYLD, and A20, these downstream effectors are not specific to TLR signaling and are targets of many other pathways. Nevertheless, they provide clues as the likely arm of TLR-signaling contributing to tumorigenesis.

### TLRs-linked to MYD88 are more frequently mutated than those linked to TRIF

In the context of cancer biology, TLR-mediated activation of MYD88 pro-inflammatory signaling is associated with tumor formation and growth, whereas activation of the Type I IFN via the TRIF adaptor has been associated with anti-tumor immunity (Lin and Karin, 2007). In accordance with those data, not only is MYD88 the most frequently mutated gene in lymphoid neoplasms, but the TLRs harboring non-silent mutations above the arbitrary threshold are those linked with MYD88 activation (TLR2 and TLR5). Interestingly, these mutations map to the extracellular domains of the TLRs and can be proposed to favor dimerization. In contrast, no non-silent mutations are reported in either TRIF, TRAM or TLR3, involved in type I IFN signaling. TLR4 leads to activation of both MYD88 and TRIF-dependent pathways. It would be interesting to check whether any of the three mutations in TLR4 favors MYD88 signaling to the detriment of TRIF signaling. These mutations could promote association with MYD88 or signaling at the cell surface instead of the endosome via decreased interaction with TRAF3 (Tseng et al., 2010). Two of the reported TRAF3 mutations are whole gene deletions and another two are premature stop codon that can be hypothesize to favor MYD88-dependent signaling as shown with RNA interference against TRAF3 (Tseng et al., 2010).

### MYD88[L265P]: one mutation to rule them all

MYD88 is the most frequently mutated gene in lymphoid neoplasm, all genes taken into account (Table 1). Moreover, one mutation dominates: the nucleotide substitution L265P. This mutant is present in 86–98% of patients with Waldenstrom's Macroglobulinemia (WM) (Treon et al., 2012; Jiménez et al., 2013; Poulain et al., 2013). However, it is not restricted to WM, with 29% of activated B-cell-like diffuse large B-cell lymphoma (ABC-DLBCL) harboring the L265P mutation, a subtype particularly difficult to cure. Overexpression of MYD88[L265P] is linked with increase cell survival and NFκB signaling (Yang et al., 2013). The mutant MYD88 activates downstream signaling via allosteric TIR-domain oligomerization (Avbelj et al., 2014) that activates TRAF6, IRAK1, and TAK1 (Ansell et al., 2014). Myddosome formation (oligomerization of MYD88 and subsequent recruitment of accessories signaling protein) is a key event linking TLR-dimerization upon ligand binding to activation of effector pathways. Therefore, if TLR-signaling contributes to lymphocyte tumorigenesis, it makes sense that one of the first step in the activation the signaling cascade is the most frequently mutated event in lymphoid neoplasms.

### CYLD and A20: negative regulator of MYD88-dependent signaling have frequent gene deletions

CYLD and A20 act as negative regulators of TLR and TNFR signaling. CYLD and TNFAIP3 are found frequently deleted in lymphoid neoplasms, which contrasts with the presence of single nucleotide substitutions more frequently found in positive regulators of MYD88 signaling (Table 1). Loss of these negative regulators would prolonged TLR-signaling and contribute to tumor growth and survival. It is important to note that it is difficult to determine whether the contribution of CYLD and A20 to tumor growth acts through TLR, TNFR, both TLR and TNFR or other pathways.

### Mutation analysis of the TLR downstream effector pathways points to activation of ERK1/2 as a target of TLR-driven lymphocyte transformation

TLR-signaling leads to the activation of four major effector pathways: the NFκB pathway and the three MAPK pathways, ERK1/2, JNK, and p38 MAPK. The process of TLR-mediated tumorigenesis may implicate all or some of these pathways. Individually, each of these pathways have been linked to cancer, and the discussion of these roles is beyond the scope of this article. In the context of TLR-signaling, emphasis has been put on NFκB activation, for both historical and technical reasons. Nevertheless, the MAPK pathways may be important contributor to tumorigenesis.

Interestingly, all of the seven genes identified carrying non-silent mutations above the 0.25% threshold that are part of the TLR effector pathways can be linked to the ERK1/2 MAPK pathway (Figure 1). This suggests that the ERK1/2 pathway is a path to tumorigenesis in TLR-driven lymphoid neoplasms. This does not rule out a role for the JNK, p38 MAPK, or NFκB, but simply mutations within these pathways are not selected during clonal selection of tumor cells. Therefore, the analysis of mutations found in the TLR network leads to the formulation of the following hypothesis: “Inhibition of ERK1/2 MAPK activation would impair lymphocytes transformation dependent on MYD88-activation.”

## Activation of ERK1/2 a critical component of the transformation process in lymphoid neoplasms?

Constitutive ERK activity is a hallmark of many B-cell malignancies (Platanias, 2003), consistent with the findings of the mutational analysis presented in Section ‘Analysis of Somatic Mutations Found in the TLR Signaling Network in Lymphoid Neoplasm’ and a whole genome sequencing study in CLL (Wang et al., 2011). Surprisingly, the upstream signals regulating ERK activation in B-cells are poorly understood, as reported in a recent article (Rickert, 2013).

### The protein kinase tumor promoting locus 2 (TPL2) is a key activator of ERK1/2 downstream of the IKK complex

Activation of ERK1/2 can occur downstream of the classical RAS-RAF-MKK1/2 pathway in response to growth factor activation (Macdonald et al., 1993). However, ERK1/2 can also be activated by another signaling pathway via the activation of the Tumor Promoting Locus 2 (TPL2, also known as MAP3K8 or COT) protein kinase (Dumitru et al., 2000). Activation of TPL2 requires phosphorylation and degradation of NFκB1 p105 by the IκB Kinase (IKK) complex (Beinke et al., 2004). Once activated TPL2 phosphorylates MKK1/2, direct upstream activators of ERK1/2. This pathway is well established as essential to ERK1/2 activation following TLR activation (Dumitru et al., 2000; Beinke et al., 2004; Banerjee et al., 2006; Rousseau et al., 2008; Martel et al., 2013). It is important to re-emphasize that following activation of TLR-signaling, ERK1/2 activation will occur in parallel to NFκB via a shared upstream activator. This means that experimental data obtained with IKKβ inhibitors where the results were solely assigned to NFκB activity may have overlooked an important contribution of the ERK1/2 MAPK cascade. Interestingly, the IKKβ [K171E] mutant identified in some lymphoid neoplasms has greater activity toward NFκB activation but has not been tested for its capacity to activate ERK1/2 (Kai et al., 2014). Based on the critical role of IKKβ in activating TPL2, it is interesting to speculate that this mutant will also lead to greater ERK1/2 activity.

### MYD88[L265P] leads to ERK1/2 activation via TPL2 in a heterologous expression system

**Figure 2:**
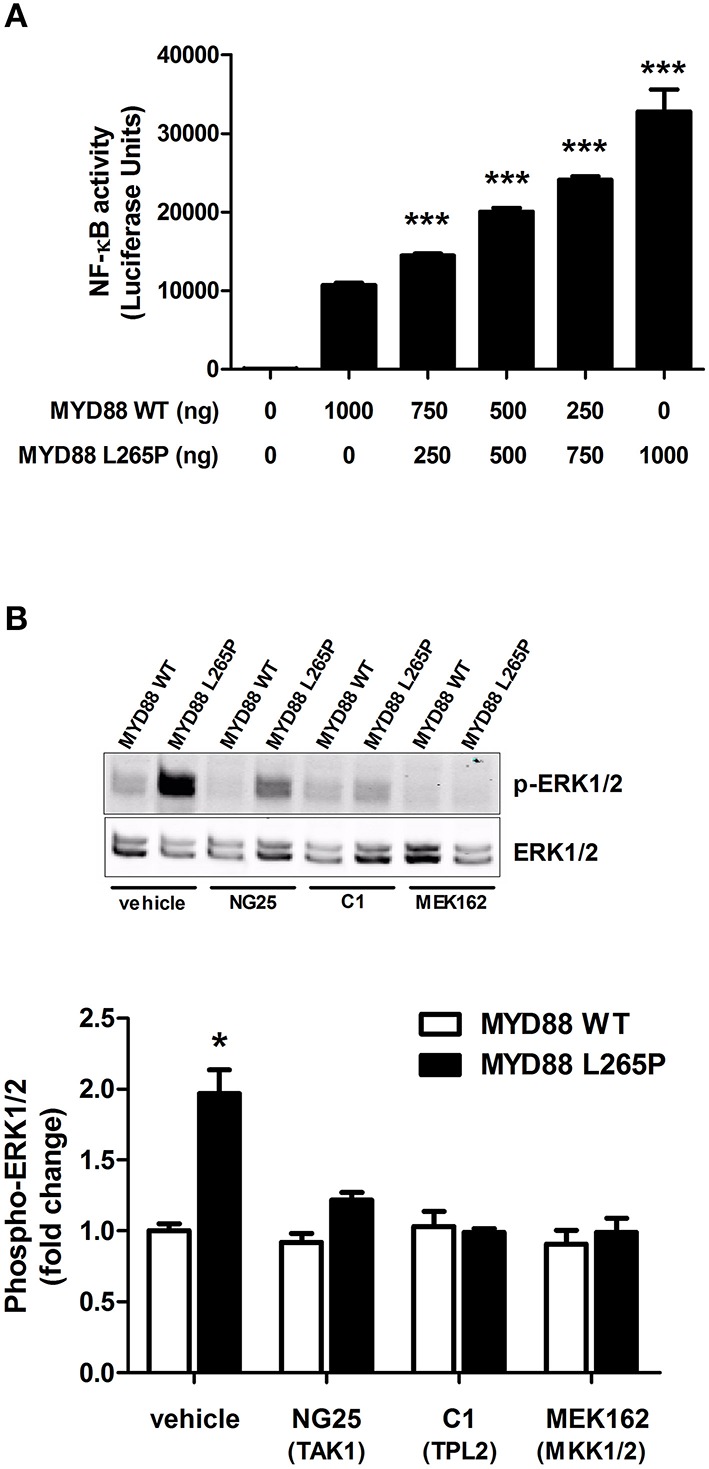
**TPL2-mediates the MYD88[L265P] ERK MAPK activation in HEK 293 cells. (A)** Cells were grown to confluence, lysed with Promega's reporter buffer and subjected to luminescence analysis as previously described. ^***^p < 0.005 vs. Myd88WT. All values are expressed as fold ± S.E.M. from four different experiments. **(B)** Cells were serum starved overnight and left untreated or pre-treated for 1 h with vehicle, NG25 (TAK1 inhibitor, 10 μM), C1 (TPL2 inhibitor, 2 μM), or MEK162 (MKK1/2 inhibitor, 1 μM). ERK1/2 phosphorylation was determined by immunoblotting. ^*^p < 0.05 vs. to Myd88WT and Myd88WT/L265P treated cells.

Heterologous expression of MYD88[L265P] in HEK293 cells, not only activates the NFκB pathway (Figure 2A), but also the ERK1/2 MAPK but only in the presence of TPL2 exogenous expression (Figure 2B). This activation can be blocked with an inhibitor of TPL2 (Figure 2B). Consistent with previously published data on NFκB activation, MYD88[L265P] leads to ERK1/2 phosphorylation in a TAK1 (MAP3K7) and MKK1/2-dependent fashion (Figure 2B).

### MKK1/2 hot spot for resistance to RAF and MEK (MKK) inhibitors

MEK inhibitors have been of great interest as novel anti-cancer agents. MEK162, showed improvement in progression-free survival of patients with metastatic melanoma (Flaherty et al., 2012) and is currently in phase II clinical trials for the treatment of myeloid leukemia. The MKK1[C121S] mutation leads to greater kinase activity and confers resistance to RAF and MEK inhibitors (Wagle et al., 2011). Similarly, the MKK2[Q60P] was found in tumor cells with sustained MAPK activation and resistance to BRAF and MEK inhibitors (Villanueva et al., 2013). Therefore, these mutations are not only oncogenic drivers but of important concern in considering treatment options due to their role in resistance to therapy.

### The complex role of ERK1/2 signaling in cellular transformation

Oncogenic ERK1/2 activation leads to its translocation to the nucleus and induction of transcription factor linked with proliferation such as FOS, JUN, and MYC (Figure 1). However, the role of ERK in tumorigenesis is not as simple as MYC-driven cell proliferation. It is highly context dependent and reflected by the fact that ERK1/2 activation is also linked with growth arrest and differentiation in both normal and transformed cells. Sustained ERK1/2 activation but not its transient activity was linked with PC12 cell differentiation (Traverse et al., 1992). Moreover, PMA-stimulation of the K562 human leukemia cell line leads to growth arrest and differentiation in a ERK1/2-dependent manner (Herrera et al., 1998). Interestingly, acute ERK1/2 hyper-activation in tumors by the oncogenic BRAF[V600E] mutant leads to tumor cell senescence (Serrano et al., 1997; Michaloglou et al., 2005). This phenomenon is not the result of ERK1/2 hyperactivity but the induction of negative signaling feedback mechanisms acting as tumor suppressors (Courtois-Cox et al., 2006). Therefore, the capacity of cells to tolerate high level of ERK1/2 activity without inducing senescence requires other transformation events, such as the loss of negative feedback regulators. Supporting this notion, acute activation of oncogenic signals in pre-B cells leads to the majority of cells dying with only a fraction progressing to malignant transformation (Shojaee et al., 2015).

In addition to its role in promoting cell proliferation when escaping senescence, ERK1/2 MAPK activation is also linked with increased cell survival and resistance to treatment like their upstream activators MKK1/2. In hairy-cell leukemia, sustained ERK1/2 activation promotes cell survival (Kamiguti et al., 2003). Sustained BCR-signaling that prolong ERK1/2 and AKT(PKB) signaling, increases the expression of the antiapoptotic protein myeloid cell leukemia-1 (Mcl-1), promoting cell survival in CLL (Petlickovski et al., 2005). Similarly, CXCR4 somatic mutations frequently found in WM lead to sustained ERK1/2 and AKT activation linked with resistance to the BTK inhibitor Ibrutinib (Cao et al., 2015).

Although no experimental data is currently available for the four ERK2 mutations identified in this analysis, it is interesting to note that the tumor samples in which they have been identified did not have mutations in MYD88 or other TLR-related signaling network component. Coexistence of MYD88 and ERK2 mutations would have undermined the theory that one of the main outcome of MYD88 activation in tumor formation is increase ERK1/2 activity.

### MYC, CBP and hnRNPA1, downstream targets of the ERK1/2 pathway with potential link with tumorigenesis

#### The ERK1/2 MAPKs are key regulators of MYC expression and function

Activation of MYC is associated with increased proliferation, more aggressive disease and poorer outcomes in Richter's Transformation, a transformation of CLL into a clonally related aggressive Diffuse Large B Cell Lymphoma (DLBCL) (Scandurra et al., 2010). B-cell Receptor (BCR)-mediated expression of MYC is regulated by ERK1/2-mediated phosphorylation of the transcription factor ELK-1 (Yasuda et al., 2008). Moreover, it has long been established that phosphorylation of MYC at Ser62 by ERK1/2 promotes cellular transformation (Pulverer et al., 1994). Therefore, the ERK1/2 MAPKs are involved at two levels of MYC regulation contributing in both cases to its transformation potential. Mutations in MYC, which also include chromosomal rearrangements, lead to increase activity that may render it independent of upstream signaling regulation. Therefore, pharmacological strategies targeting upstream MYC signaling in cells harboring MYC activation are hypothesized to be less effective than treatments inducing increase cell death via antagonizing for example BCL-2 that cooperates with MYC in transformation of pre-B cells (Vaux et al., 1988).

#### Loss of CBP function increases ERK1/2 tumorigenic potential

CBP act as a transcriptional co-activator for numerous transcription factors, including many oncogenes. In addition, it can also act as an histone acetylase with p300 (another gene frequently mutated in cancer) to dynamically regulate gene expression. It is tempting to hypothesize that based on its interaction with TFs known to be oncogenes, CBP promotes tumor formation. However, most evidences point to CBP as a tumor suppressor. This has been clearly demonstrated in mice, where CBP heterozygotes developed hematologic malignancies that were associated with the loss of the second CBP allele (Rebel et al., 2002). Moreover, patients suffering from Rubinstein-Taybi Syndrome, associated with mutations and deletions in CBP, have an increased risk for leukemia (Jonas et al., 1978).

Interestingly, during persistent ERK1/2 activation, CBP associates with MAPKAP-K1 (p90RSK) to promote growth arrest and cell differentiation (Wang et al., 2003). Accordingly, in the analysis presented in this paper, CBP is found frequently deleted, which could be a permissive event in presence of persistent ERK1/2 signaling to promote cellular growth instead of arrest. This idea is supported by a study investigating relapsed acute lymphoblastic leukemia (ALL). Non-synonymous somatic mutations in CBP were found in conjunction with RAS signaling pathway mutations within the same tumor samples (Mullighan et al., 2011). The CBP mutations identified impaired histone acetylation and transcriptional activity of CBP. These results not only support the idea that loss of CBP activity enhances ERK1/2-mediated transformation, but lend further strength to the idea presented above about the importance of secondary events impairing high or persistent ERK1/2 activation-induced senescence.

#### hnRNPA1 expression and phosphorylation occurs downstream of ERK1/2 activation

In T lymphocytes, hnRNPA1, a TNFα AU-rich element binding protein (Rousseau et al., 2002) is phosphorylated by MNK1, a protein kinase activated by ERK1/2 and p38 MAPK (Buxadé et al., 2005). Phosphorylation of hnRNPA1 contributes to post-transcriptional regulation of TNFα (Buxadé et al., 2005). Interestingly, recent evidences have highlighted roles for genes involved in post-transcriptional regulation, such as mRNA splicing, as oncogenic driver in CLL (Wang et al., 2011). Moreover, MYC has been shown to increase hnRNPA1 expression leading in turn to higher expressing of pyruvate kinase, contributing to aerobic glycolysis frequently observed in tumor cells (David et al., 2010). In support for a role of hnRNPA1 in lymphoid neoplasms, aberrant expression of hnRNPA1 was described in acute leukemia (Choi et al., 2014). Therefore, increased expression of hnRNPA1 driven by MYC and its phosphorylation by MNK1 (two ERK1/2-dependent events), or its aberrant expression resulting from gain-of-function mutations, would increase pro-inflammatory gene synthesis and aerobic glycolysis to contribute to tumorigenesis.

## Conclusions

### TLR-MYD88-IKKβ-TPL2-MKK1/2-ERK1/2, a key path cell to transformation?

Previously published experimental data have established the important contribution of MYD88-mediated signaling in a number of B-cell malignancies (Ngo et al., 2011; Treon et al., 2012; Wang et al., 2014). This is further supported by the mutational analysis presented in this hypothesis and theory article. Moreover, by looking at the frequency of gene mutations downstream of MYD88 activation, the ERK1/2 MAPK pathway is highlighted as a potential key effector pathway of tumorigenesis via activation of downstream targets such MYC and hnRNPA1. The overall outcome of this TLR-mediated signaling would be at least two fold: (1) increased in cell proliferation via MYC activation and anaerobic glycolysis (2) enhanced pro-inflammatory signaling, in particular the expression of TNFα, which would contribute to promote changes in the tumor microenvironment favoring tumor growth.

### Limitations of the analysis

There are a number of limitations to the current analysis. First, for a number of mutations identified, no experimental data is available to support their role in modifying TLR signaling. Moreover, these mutations were mostly considered in isolation from each other. But as discussed in the ERK1/2 and CBP sections, tumor samples have multiple mutations that may act together in order to promote tumorigenesis. Furthermore, lymphoid neoplasms are numerous, affecting T and B lymphocytes at different stages of their differentiation. Some factors play more important roles in one disease over the other, such as MYD88[L265P] in WM, or in pre-B cells vs. mature B cells (Rickert, 2013). Finally, mutations may have different roles in the tumor microenvironment vs. cells grown in tissue culture. The tumor microenvironment is likely playing an important role in understanding the breadth of impact that inflammation has on tumorigenesis, particularly related to TLR-mediated pro-inflammatory signaling.

### Testing the hypothesis in waldenstrom's macroglobulinemia

A number of hypothesis have been formulated along the way, but the overarching one would be that inhibition of ERK1/2 MAPK activation would prevent tumor growth downstream of MYD88[L265]. In the context of MYD88 driven tumorigenesis, a particularly attractive target would be the protein kinase TPL2, which should abrogate excess ERK signaling. Pharmacological inhibitors have been developed that target TPL2 as shown in Figure 2B, and it would be highly interesting to test these in a more physiologically relevant setting than an heterologous expression system. Since WM tumor cells have an overwhelming presence of MYD88[L265P] mutations, they represent an excellent model to test the hypothesis put forth in this article.

## Experimental procedures

### Materials

The TAK1 inhibitor NG25 and the TPL2 inhibitor Compound 1 were kindly provided by Professor Sir Philip Cohen (MRC PPU, University of Dundee, UK). The MKK1/2 inhibitor MEK162 (Binimetinib) was purchased from Selleck Chemicals (Houston, TX, USA).

### ERK1/2 immunoblotting

100,000 HEK293TLR5 cells (Invivogen, San Diego, USA, #HKB-HTLR5) were seeded in a 24-wells plate and transfected with 200 ng of pCDNA3.1-TPL2 and 800 ng of pCDNA3.1-Myd88 or the empty vector for 48 h using polyethylenimine (PEI). Cells were grown to confluence, lysed and lysates were subjected to SDS-PAGE. Quantitative analysis graph was obtained with the signal intensity of an antibody recognizing the phosphorylated forms of ERK1/2 at Thr202/Tyr204 normalized to the signal obtained with antibody that recognizes all forms of ERK1/2.

### NFκB luciferase assay

Cells were transfected with 200 ng of pGL4.28-NF-κB and a combination of varying amounts of pCDNA3.1-Myd88 WT and/or pCDNA3.1-Myd88 L265P for a total of 1000 ng of DNA per transfection. Cells were grown to confluence, lysed with Promega's reporter buffer and subjected to luminescence analysis.

## Author contributions

SR and GM have made substantial contributions to the conception, design, acquisition, analysis and interpretation of data for the work. SR has drafted the work and revised it critically for intellectual content. SR and GM have approved the final version to be published and agreed to be accountable for all aspects of the work.

## Funding

We acknowledge the financial support of Canadian Institute of Health Research (MOP#123496). The Meakins-Christie Laboratories—MUHC-RI, are supported by a Centre grant from Les Fonds de Recherche du Québec-Santé (FRQ-S). SR acknowledges a salary award from the FRQ-S.

### Conflict of interest statement

The authors declare that the research was conducted in the absence of any commercial or financial relationships that could be construed as a potential conflict of interest.

## Supplementary material

Supplementary Figure 1 **TLR signaling network assembled for mutation analysis.** Schematic representation of TLR5/TLR5 and TLR2/TLR6 MYD88-dependent intracellular signaling network built using CellDesigner. The network comprises 77 components, that were assembled based on the current literature (refer to text for references). LP stands for di-acylated lipopeptides; circled “P” denotes phosphorylation; circled “Ub” denotes ubiquitination; Ub(K63) denotes Lysine 63 poly-ubiquitin chains; Ub(Met-1) denotes linear ubiquitin chains; black lines, denote positive signal flow; red lines, denote negative regulatory events; the color purple highlights some commonly used inhibitors.
