# Supplementary figures and images for: Gain-of-Function Mutations in the Toll-Like Receptor Pathway: TPL2-Mediated ERK1/ERK2 MAPK Activation, a Path to Tumorigenesis in Lymphoid Neoplasms?
Source: Front Cell Dev Biol. 2016 May 26;4:50. doi: 10.3389/fcell.2016.00050 (PMC4881378; doi:10.3389/fcell.2016.00050)

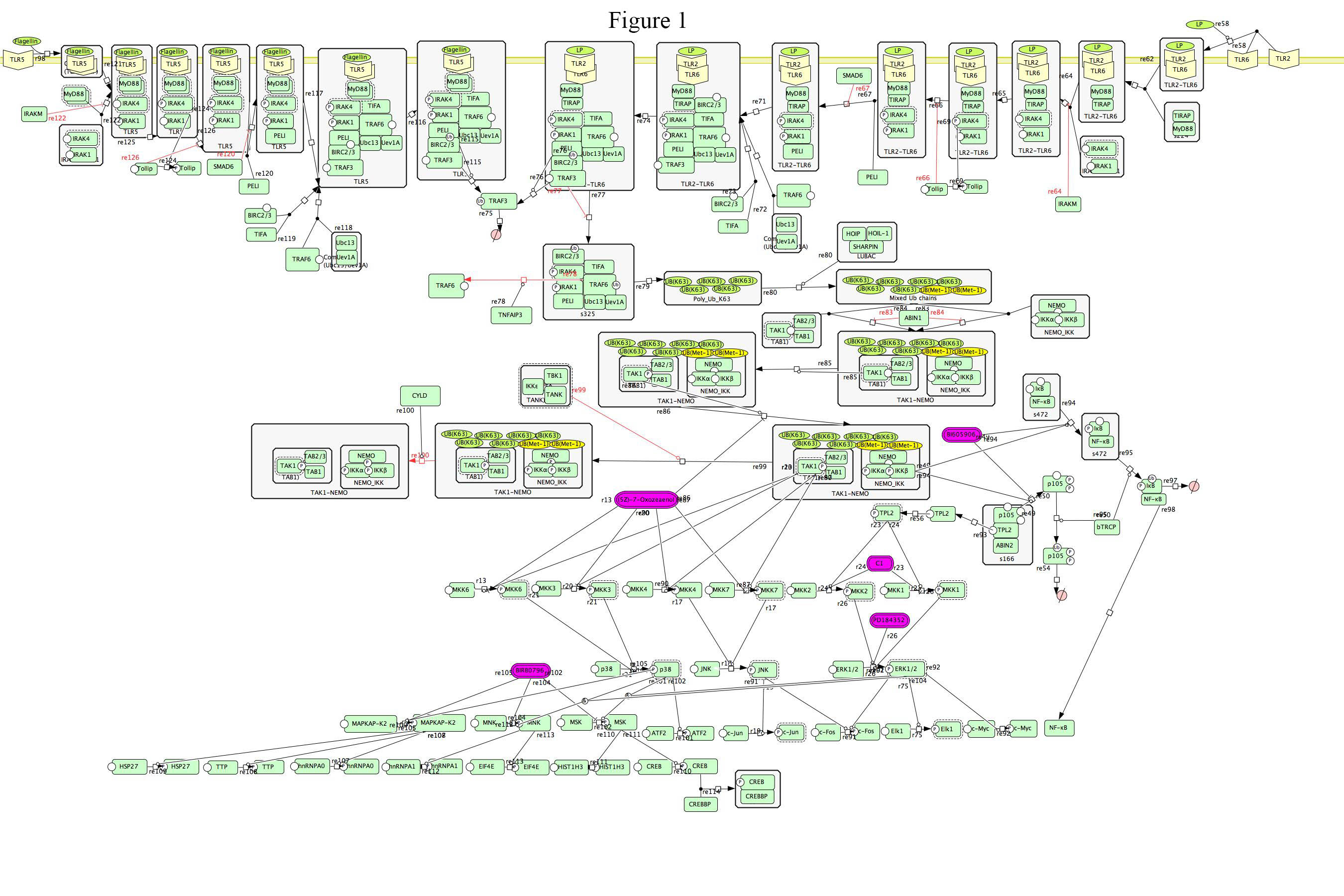

Supplement: Supplementary Figure 1 — TLR signaling network assembled for mutation analysis. Schematic representation of TLR5/TLR5 and TLR2/TLR6 MYD88-dependent intracellular signaling network built using CellDesigner. The network comprises 77 components, that were assembled based on the current literature (refer to text for references). LP stands for di-acylated lipopeptides; circled “P” denotes phosphorylation; circled “Ub” denotes ubiquitination; Ub(K63) denotes Lysine 63 poly-ubiquitin chains; Ub(Met-1) denotes linear ubiquitin chains; black lines, denote positive signal flow; red lines, denote negative regulatory events; the color purple highlights some commonly used inhibitors. [file Image1.JPEG]
